# Process Analytical Chemistry

**DOI:** 10.6028/jres.093.018

**Published:** 1988-06-01

**Authors:** Bruce R. Kowalski

**Affiliations:** Center for Process Analytical Chemistry, Department of Chemistry BG-10, University of Washington, Seattle, WA 98195

Chemical analysis can be thought of as a means to obtain chemical information on a chemical system or process. Traditionally, the system or process is sampled and the samples are transported to the analytical laboratory where analytical procedures and instruments are used to generate data which are then converted to chemical information by calibrated mathematical models. Unfortunately, this valuable information is not used or needed in the laboratory. Further, it is currently being recognized that sampling errors and time delays associated with sample transport and analysis make it nearly impossible to control complex chemical processes with the required degree of success. Traditionally, chemical engineers have relied predominantly on pressure, temperature, and flow sensors to monitor and control their processes. More recently, there have been increasing attempts to make laboratory instruments “process hardened” and move from “off-line” to “at-line” analysis. In addition, there has been a growing demand for the development of novel sensors to allow for true “on-line” analysis and control (and also the development of noninvasive sensors for some problematical process applications). Process Analytical Chemistry [1] seeks to create new sensors and analytical instruments that can be used as integral parts of a wide range of chemical processes for process monitoring and control.

The Center for Process Analytical Chemistry (CPAC) at the University of Washington was founded as a University/Industry Cooperative Research Center established by a grant from the National Science Foundation. CPAC serves as a focus for basic research in process analytical chemistry as well as a clearinghouse for information on analytical methods and a training ground for scientists and engineers skilled in on-line chemical processing, monitoring and control.

Trace analysis in process analytical chemistry has not received the same emphasis as it has, say, in environmental or health sciences. More often than not, process engineers would prefer to follow the concentrations of major reaction products for on-line control. This may be because new process analytical sensors and instrumentation capable of trace analysis have not met the severe tests required for use with process streams. CPAC researchers are working on new methods and applications designed to contribute to improved accuracy in trace, minor and major component analysis. Among these contributions are included chemometric methods for designing optimal arrays of sensors [2] and characterizing performance as they are developed [3]. The characterization of sensor arrays, based on the concept of net analyte signal [4], allows analytical chemists to estimate the sensitivity, signal to noise ratio, selectivity, and limits of determination for individual analytes. Using these statistics embedded in an expert system being assembled at CPAC, the analyst will be able to construct sensor arrays or select wavelengths that will meet the accuracy requirements for given analytes in a variety of sample matrices.

The use of nonselective arrays of sensors or multiple wavelengths is important for process analytical chemistry where the development of fully selective sensors is often too costly. For this reason, CPAC has supported work on new multivariate calibration [5] methods and has even provided a theoretical foundation for partial least squares [6], a method enjoying popularity in industrial applications. Previously, the use of these multivariate methods had the disadvantage that predicted analyte concentrations on real samples were not accompanied by confidence level estimates. This is because the three sources of error (calibration sensor responses, “known” concentrations, and sensor responses from the unknown samples) combined in a very complex manner. In view of the importance of accuracy in process modelling and control, CPAC researchers have developed a method to estimate accurately the variance of concentrations predicted from multivariate calibration models that include all sources of error [7]. The method separates bias from precision and can therefore be used as an estimate of accuracy.
